# An Administrative Data-based Surrogate Definition Identifies Children Evaluated Beyond Physical Examination for Suspected Appendicitis

**DOI:** 10.1097/pq9.0000000000000343

**Published:** 2020-10-23

**Authors:** Eric W. Glissmeyer, Sydney Ryan, Nanette C. Dudley, Jeff E. Schunk, Jeremy Nielsen, Cindy Weng, David E. Skarda

**Affiliations:** * Department of Pediatrics, University of Utah, Salt Lake City, Utah; † Intermountain Healthcare, Salt Lake City, Utah; ‡ Western Governor’s University, Salt Lake City, Utah; § Department of Surgery, University of Utah, Salt Lake City, Utah.

## Abstract

Previous studies have reported on the evaluation of patients diagnosed with appendicitis. Very little is known about all patients evaluated for suspected appendicitis. Patients evaluated beyond physical examination with laboratory and imaging testing, then found not to have appendicitis, are more difficult to identify. Data readily available in administrative databases may be used to identify these patients.

**Methods:**

A multidisciplinary team developed a surrogate definition for evaluating suspected appendicitis in children based on available administrative data. Appendicitis was “suspected” if the patient underwent ultrasonography of the appendix or had a chief complaint of abdominal pain with both complete blood count performed and the word “appendicitis” in the ED provider note. Performance characteristics described the surrogate definition’s ability to retrospectively identify patients evaluated for suspected appendicitis through comparison to a population identified via chart review.

**Results:**

Compared with manual chart review of 498 patients from June 2014, the surrogate definition identified patients evaluated beyond physical examination for suspected appendicitis with a sensitivity of 79.8%, a specificity of 96.3%, a positive predictive value of 83.3%, and a negative predictive value of 95.3%. Of the 94 patients evaluated beyond physical examination for suspected appendicitis, 37 (39%) underwent appendectomy.

**Conclusions:**

Health systems can retrospectively identify children evaluated beyond physical examination for appendicitis using discrete administrative data and a word search of clinical notes. This surrogate definition for evaluation of suspected appendicitis enables research in quality improvement efforts and health care resource utilization.

## INTRODUCTION

Appendicitis is the most common surgical emergency in children,^1^ but relatively little is known about resource utilization during the evaluation of patients suspected of having appendicitis. Various identifiers have been used to retrospectively search for patients evaluated for appendicitis, including diagnostic imaging tests,^2–4^ hospital admission,^5^ or the use of diagnostic algorithms^6,7^ in the electronic health record (EHR). However, not all patients evaluated for suspected appendicitis beyond physical examination by a physician can be retrospectively identified with high specificity by chief complaints, diagnoses, laboratory tests, or imaging tests highly specific for appendicitis (like a focused diagnostic ultrasound of the vermiform appendix). Therefore, a retrospective method for identifying patients who underwent evaluation for appendicitis beyond physical examination is necessary to enable the study of health care resource utilization in this population.

Others have used prospective study methods with research assistants in the emergency department (ED) to identify patients evaluated for suspected appendicitis.^8,9^ While powerful, these efforts require significant human effort and real-time surveying of busy clinical caregivers to understand intent. However, prospective studies capture clinical data (such as history and examination findings) not easily queried retrospectively from databases. Using only chief complaints of abdominal pain to define a population of patients evaluated for suspected appendicitis requires manual chart review.^10^ Manual chart review also requires a significant time commitment from staff with adequate clinical expertise. Over time, this commitment must be repeated each time utilization review, research, or quality improvement efforts are conducted on different patients.

Hospital administrative data are collected during routine hospital operations. With near-universal use of an EHR, these data are readily available. Natural language analytics are increasingly utilized to identify patients with specific characteristics from free-text documentation.^11^ A retrospective method using available administrative data and EHR capabilities could provide a reusable tool for retrospective insight into physicians’ practice patterns evaluating patients for suspected appendicitis.

Our objectives were to develop an administrative data-based surrogate definition to identify patients who received diagnostic evaluation beyond physical examination for suspected appendicitis for quality improvement efforts and health care resource utilization review. We also describe the performance characteristics of this surrogate definition’s ability to identify these patients compared with manual chart review retrospectively.

## METHODS

**Fig. 1.**
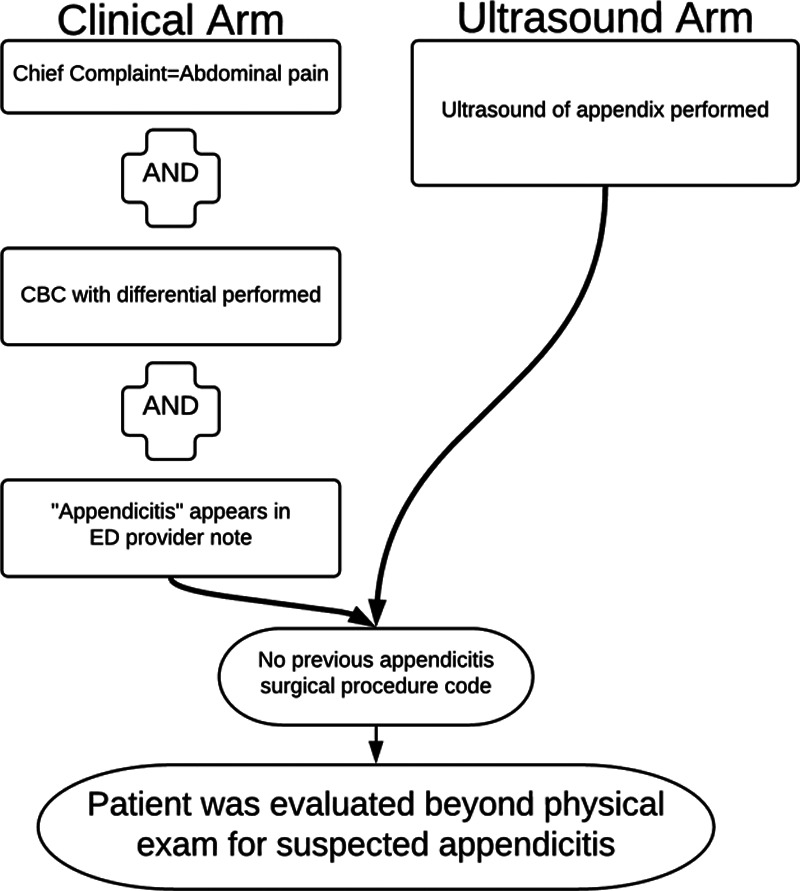
Design of surrogate definition for identifying patients evaluated for appendicitis beyond physical examination in the emergency department.

The institutional review boards of the University of Utah and Intermountain Healthcare (Salt Lake City, UT) approved this study and granted a waiver of informed consent. The study was conceived and carried out at Primary Children’s Hospital, a level 1 trauma and regional referral center managed by Intermountain Healthcare with an ED staffed by pediatric emergency medicine faculty of the University of Utah. The authors met with general surgery, radiology, and pediatric emergency medicine colleagues. They used a modified Delphi-method to develop a surrogate definition for suspected appendicitis based on chief complaint, laboratory study, diagnostic right lower quadrant ultrasound, and free-text data available in the Intermountain Healthcare electronic data warehouse administrative database. The output of this initial effort was an administrative data-based surrogate definition for suspected appendicitis shown in Figure 1. Appendicitis was “suspected” if the patient underwent an ultrasound of the appendix or had a chief complaint of abdominal pain with both complete blood count with differential performed and the word “appendicitis” in the ED provider note. The surrogate definition identifies patients as not suspected if an appendectomy procedure was performed previously, as identified in our case-mix diagnoses table by surgical procedure codes (ICD-9 47.01, 47.09, 47.11, 47.19). The database query was performed in the Intermountain Healthcare electronic data warehouse using SQL v2016 and queried for ED chief complaint, complete blood count lab test codes, focused appendix ultrasound studies performed, and search for the word “appendicitis” in ED physician notes at Primary Children’s Hospital (Fig. 1). Limiting the query to data from Primary Children’s Hospital was consistent with the single-institution focus at that time of quality-improvement and resource utilization efforts.

### Testing of Surrogate Definition

To determine the surrogate definition’s performance characteristics, 1 of the study authors (S.R.) performed an initial detailed retrospective review of 498 encounters out of a total of all 3,313 ED encounters at our facility in June 2014. This review was validated by the senior author (EG) performing a detailed review of 96 of the 498, among which no disagreement was identified. The authors (S.R. and E.W.G.) classified these patients as “evaluated for suspected appendicitis” beyond physical examination and “not evaluated for suspected appendicitis” beyond physical examination based upon the diagnostic workup performed, documented physical exam findings, and documented assessments in medical decision making portions of the ED note.

### Inclusion Criteria

The authors selected these 498 encounters for manual review because their chief complaints and disposition diagnoses indicated that the treating physician could have evaluated the patient for appendicitis, beyond physical examination. Table 1 displays the chief complaints and principal disposition diagnoses of these 498 patients. The authors then applied the surrogate definition for suspected appendicitis to these 498. The authors did not exclude patients with chronic diseases like inflammatory bowel disease, as these patients may also have acute appendicitis. Nor did the authors exclude patients of young age (ie, <4 years) in whom appendicitis diagnosis is challenging and less frequently identified compared with older children because the surrogate definition would be intended to be applied to these patients as well.

**Table 1.** Chief Complaints and Principal Disposition Diagnoses of 498 Emergency Department Encounters From June 2014

Chief complaint	Frequency, n (%)
Abdominal pain	257 (51.6)
Vomiting	203 (40.8)
Fever	21 (4.2)
Other (dehydration, anorexia, crying, diarrhea, flank pain, back pain)	17 (3.4)
Principal disposition diagnosis	Frequency, n (%)
Abdominal pain	129 (25.9)
Vomiting	96 (19.3)
Gastroenteritis	63 (12.6)
Other*	42 (8.5)
Dehydration	37 (7.5)
Acute appendicitis	32 (6.4)
Fever	28 (5.6)
Constipation	22 (4.4)
Diarrhea	14 (2.8)
Viral syndrome	8 (1.6)
UTI	6 (1.2)
Intussusception	5 (1.0)
Bowel obstruction	4 (0.8)
Gastritis	4 (0.8)
Hydrocephalus	4 (0.8)
Pyloric Stenosis	4 (0.8)

*Other: strep pharyngitis, dyspepsia, flank pain, otitis media, pneumonia, pyelonephritis, chronic abdominal pain, bowel perforation, shunt malfunction, pneumoperitoneum, ureteral calculus, new-onset diabetes, meningitis, altered mental status, mesenteric hematoma, hepatitis, splenic laceration, postoperative complications.

### Exclusion Criteria

The authors excluded from the chart review 2 subjects with a history of a previous appendectomy. These cases would be likewise excluded by the physician performing a real-time evaluation for acute abdominal pain. The surrogate definition also is designed to exclude these patients.

Analysis of column and row totals determined the extent of the surrogate definition’s agreement with chart review of whether patients were evaluated for suspected appendicitis beyond physical examination.

## RESULTS

Manual chart review of 498 unique ED encounters identified 94 children with abdominal pain evaluated for suspected appendicitis beyond the physical exam, and 404 children not evaluated for suspected appendicitis. Of the 94 patients evaluated for suspected appendicitis, 37 (39%) underwent appendectomy. The surrogate definition retrospectively applied to these 498 encounters correctly identified 75 of 94 patients evaluated for suspected appendicitis (sensitivity 79.8%) and correctly identified 389 of 404 patients not evaluated for suspected appendicitis (specificity 96.3%). The surrogate definition’s positive predictive value was 83.3%, and the negative predictive value was 95.3% (Table 2). Of the 90 identified positively by the surrogate definition, 50 were identified by the ultrasound arm (Fig. 1) and 40 by the clinical arm.

**Table 2.** Performance Characteristics of the Surrogate Definition to Identify Patients as Evaluated Beyond Physical Examination for Suspected Appendicitis

	Evaluated for Suspected Appendicitis	Not Evaluated for Suspected Appendicitis
Surrogate definition positive	75	15
Surrogate definition negative	19	389
Statistic	Value (%)	95% Confidence Interval
Sensitivity	79.8	0.72–0.88
Specificity	96.3	0.94–0.98
Positive predictive value	83.3	0.76–0.91
Negative predictive value	95.3	0.93–0.97

## DISCUSSION

Health systems can retrospectively identify patients evaluated beyond physical examination for appendicitis using a surrogate definition, discrete administrative data, and a word search of clinical notes. This approach may enable quality improvement efforts and health care resource utilization review for the diagnosis of appendicitis beyond physical examination.

We believe the sensitivity of our surrogate definition at 79.8% does not detract from its usability in resource utilization and quality improvement efforts. In these efforts generally, the need for a dataset with high specificity (96.3%) outweighs the desire for optimal sensitivity. Furthermore, many of the cases the surrogate definition failed to identify were transferred, thus not the population of most significant interest when studying resource utilization in evaluation for suspected appendicitis at one’s facility. Our facility is a referral center that receives many of our patients from transferring EDs. The majority (12 of 19) of the patients evaluated for suspected appendicitis not identified by the surrogate definition were transferred from outside facilities. The surrogate definition did not identify them (false-negatives in the sensitivity analysis) because it included laboratory tests and appendix ultrasound tests ordered only from our facility. In our practice, we do not routinely repeat studies performed from outside facilities, especially if found to be conclusive and consistent with the patient’s physical exam and presentation at our facility. If one wanted to evaluate a multi-hospital health system’s care of patients evaluated for suspected appendicitis, a surrogate definition could identify a population with improved sensitivity if it were to be applied to administrative data from the transferring hospitals as well. This approach may be difficult for any hospital like ours, which receives transfers from hospitals both part of Intermountain Healthcare using our same EHR and from other hospitals not part of our system that use various other EHRs.

The surrogate definition included both a clinical arm and an ultrasound arm for multiple reasons. Not all patients at our institution receive an ultrasound of the appendix. Some patients receive imaging tests before being transferred, and some patients with PAS scores ≥8 are taken to surgery based on physical examination and laboratory testing alone. However, the ultrasound arm is crucial because, in our system, a focused ultrasound of the appendix is increasing in use as the only diagnostic test in evaluating patients for suspected appendicitis.^12^ Local practice patterns (such as the use of a primary imaging modality for suspected appendicitis other than ultrasound) or changing patterns over time (if another imaging modality^13^ were to become commonly used to diagnose appendicitis) would dictate the need for nuanced alteration of the surrogate definition. As for the patients in this study, 50 of the 90 identified by the surrogate definition were evaluated for suspected appendicitis beyond physical examination and received an ultrasound at our institution. In the development of the surrogate definition clinical arm (Fig. 1), the authors questioned the need to include the criterion of the word “appendicitis” appearing in the ED physician note. However, removing this criterion results in an increase of false-positive results and a resultant drop in specificity from 96% to 75%. Therefore, the surrogate definition retains this chart text data criterion. We did not search for variations on the word “appendicitis” because medical transcriptionists exclusively transcribed our clinical notes, and typed variations or errors in recognition software did not apply. Including this criterion in our data, today would require a more expansive search terminology.

This study’s strengths include a manual chart review to establish the reference population of patients evaluated and not evaluated for appendicitis beyond the physical exam. During the study period, provider staffing (attending physician, fellow, and nurse practitioner) was consistent. Also, no changes occurred during this study period in the availability of factors that may influence the evaluation of suspected appendicitis, including 24 hours per day appendix ultrasound and in-house surgical consultation. Limitations include those inherent in administrative database research and retrospective chart review in determining whether patients were evaluated beyond physical examination for suspected appendicitis. However, these limitations were minimized by the consistent laboratory, chief complaint, and ultrasound codes during the study period. Chief complaints of abdominal pain alone are nonspecific and not highly sensitive^14^ for whether a patient was evaluated for suspected appendicitis (Table 1).

Nonetheless, chief complaints are among the few commonly available administrative data that can be used to identify populations of patients suspected of having a disease when no specific and universally utilized test exists. In a hospital or system that utilizes CT primarily as the initial diagnostic test for appendicitis, the surrogate definition may need to include that in addition to, or in place of the ultrasound arm.

The use of simple word search methods for the word “appendicitis” in the clinical notes does not discern meaning. A further improvement could be utilizing natural language processing methodology (ie, appendicitis was suspected versus was not suspected). We did not go this additional step because, with specificity 96.3%, the gains of a natural language processing approach were assessed to be minimal.

## CONCLUDING SUMMARY

Children with abdominal pain who receive an evaluation for suspected appendicitis beyond physical examination in the ED can be retrospectively identified with high specificity using an administrative data-based surrogate definition. This method may empower research and quality improvement efforts measuring health care utilization in these patients, such as trends in imaging study use and hospital admission after application of diagnostic algorithms for children with acute abdominal pain.

## ACKNOWLEDGMENTS

Assistance with the study: we thank the emergency department staff and patients of Primary Children’s Hospital and support of Intermountain Healthcare.

## DISCLOSURE

The authors have no financial interest to declare in relation to the content of this article.

## References

[1] Rentea RM St Peter SD Pediatric appendicitis. Surg Clin North Am. 2017; 97:93–112 27894435 10.1016/j.suc.2016.08.009

[2] Skarda DE Rollins M Andrews S One hospital, one appendectomy: the cost effectiveness of a standardized doctor’s preference card. J Pediatr Surg. 2015; 50:919–922 25805009 10.1016/j.jpedsurg.2015.03.009

[3] Zoarets I Poluksht N Halevy A Does selective use of computed tomography scan reduce the rate of “white” (negative) appendectomy? Isr Med Assoc J. 2014; 16:335–337 25058992

[4] Blitman NM Anwar M Brady KB Value of focused appendicitis ultrasound and Alvarado Score in predicting appendicitis in children: can we reduce the use of CT? AJR Am J Roentgenol. 2015; 204:W707–W712 26001260 10.2214/AJR.14.13212

[5] Thompson GC Schuh S Gravel J; Pediatric Emergency Research Canada. Variation in the diagnosis and management of appendicitis at Canadian pediatric hospitals. Acad Emerg Med. 2015; 22:811–822 26130319 10.1111/acem.12709

[6] Depinet H Allmen D Von Towbin A Risk stratification to decrease unnecessary diagnostic imaging for acute appendicitis. Pediatrics. 2016; 138:e20154031 27553220 10.1542/peds.2015-4031

[7] Scheller RL Depinet HE Ho ML Utility of pediatric appendicitis score in female adolescent patients. Acad Emerg Med. 2016; 23:610–615 26824846 10.1111/acem.12916

[8] Fleischman RJ Devine MK Yagapen MA Evaluation of a novel pediatric appendicitis pathway using high- and low-risk scoring systems. Pediatr Emerg Care. 2013; 29:1060–1065 24076607 10.1097/PEC.0b013e3182a5c9b6

[9] Saucier A Huang EY Emeremni CA Prospective evaluation of a clinical pathway for suspected appendicitis. Pediatrics. 2014; 133:e88–e95 24379237 10.1542/peds.2013-2208

[10] Kharbanda AB Madhok M Krause E Implementation of electronic clinical decision support for pediatric appendicitis. Pediatrics. 2016; 137:e20151745 27244781 10.1542/peds.2015-1745

[11] Norman B Davis T Quinn S Automated identification of pediatric appendicitis score in emergency department notes using natural language processing. Paper presented at: 2017 IEEE EMBS International Conference on Biomedical and Health Informatics, BHI, February 16, 2017, Orlando, Fla

[12] Cundy TP Gent R Frauenfelder C Benchmarking the value of ultrasound for acute appendicitis in children. J Pediatr Surg. 2016; 51:1939–1943 27670963 10.1016/j.jpedsurg.2016.09.009

[13] Kearl YL Claudius I Behar S Accuracy of magnetic resonance imaging and ultrasound for appendicitis in diagnostic and nondiagnostic studies. Acad Emerg Med. 2016; 23:179–185 26765503 10.1111/acem.12873

[14] Drapkin Z Dunnick J Madsen TE Pediatric appendicitis: association of chief complaint with missed appendicitis. Pediatr Emerg Care. 2020; 36:e204–e207 29324631 10.1097/PEC.0000000000001390

